# No Intentions in the Brain: A Wittgensteinian Perspective on the Science of Intention

**DOI:** 10.3389/fpsyg.2019.00946

**Published:** 2019-04-26

**Authors:** Annemarie Kalis

**Affiliations:** Utrecht University, Utrecht, Netherlands

**Keywords:** intentions, action, causal theory of action, functionalism, Wittgenstein, folk psychology

## Abstract

In their paper “Why we may not find intentions in the brain,” Uithol et al. (2014) convincingly argue that “the processes underlying action initiation and control are considerably more dynamic and context sensitive than the concept of intention can allow for.” Their paper could be seen as a critical note to the widespread tendency to search for identifiable neurocorrelates of mental concepts. Their more specific suggestion is that the absence of clear neural correlates undermines the traditional understanding of intention. In this paper I will try to take their argument a step further. First of all, I will argue that our folk psychology leaves room for various understandings of intentions, and that the concept of intention discussed by Uithol et al. is an academic concept that has its roots in the causal theory of action and in functionalist approaches to cognition. I will argue that both these paradigms are contested, and that there seems to be theoretical wiggle room for alternative understandings of intention. Subsequently I outline such an alternative perspective based on Wittgensteinian philosophy of psychology, emphasizing the regulative role of intention talk. However, the proposed understanding raises the question how to think about neural realization: is intention talk “just” talk, or do intentions really exist? I will propose that intention talk should be understood as a form of pattern recognition, and that the patterns involved are extended in both space and time. The conclusion outlines some important implications for the neuroscientific investigation of intentions.

## Introduction

This paper is about the philosophical question of how to understand the concept of *intention.* How can we make sense of the phenomenon that we can commit ourselves to certain courses of action (such as learning Chinese, visiting one’s parents or buying a new bicycle), and that such commitment actually makes a difference? Although the question how to understand intention is a philosophical question, this question has recently become urgent also for psychologists and neuroscientists. During the last 30 years, cognitive science has produced an increasing amount of studies investigating the neural underpinnings of intention formation or conscious volition (Libet, 1985; Blakemore and Decety, 2001; Haggard and Libet, 2001; Haggard and Clark, 2003; Haynes et al., 2007; Bisley and Goldberg, 2010; Gallivan et al., 2011;Haynes, 2011). Most of these build on Libet’s seminal research, which showed that the rise of a conscious intention to act is preceded by so-called readiness potentials (Libet, 1985). These studies already worked with a certain implicit understanding of what intentions are. In Libet’s experimental setup (and in many of its spin offs), conscious intention and its timing are measured by means of self-report: participants are required to report on the precise moment at which they formed an intention to act in each trial. More precisely, they are asked to report on the first appearance of “conscious awareness of wanting to perform” the required behavior. “The experience was described as an ‘urge’ or ‘decision’ or ‘intention’ to move, though subjects usually settled for the words ‘wanting’ or ‘urge’” (Libet et al., 1983). Libet justifies this method as follows: “if a conscious intention or decision to act actually initiates a voluntary event, then the subjective experience of this intention should precede or at least coincide with the onset of the specific cerebral processes that mediate the act” (Libet, 1985, p. 529). This shows that for Libet, (1) an intention is necessarily conscious, (after all, report of conscious awareness is taken to be decisive evidence for the presence of an intention), and (2) intentions are thought to play a causal role in the bringing about of voluntary actions. What Libet took himself to have shown, is that intentions should not be seen as *unmoved movers* because voluntary actions are already initiated before the formation of a conscious intention *–* a conclusion which is often taken to have widespread implications for the free will debate.

In response to Libet’s work, another line of research has come to analyze different kinds of preparatory neural processes not as *precursors* of conscious intentions, but as their neural *underpinnings* (nowadays usually not by measuring readiness potentials, but by employing sophisticated analyses of fMRI signals such as multivoxel pattern analysis (MVPA). For example, several papers bear titles referring to the “reading” or “decoding” of intentions from brain activity (Gallivan et al., 2011; Haynes, 2011). The proclaimed aim of such studies is to increase insight in the “neural underpinnings of highly cognitive and abstract processes such as intentions” (Gallivan et al., 2011, p. 9599). What has often been shown by now, is that “extended regions of cortex prepare for upcoming movements” (Haynes, 2011, p. 11). In these studies, the notion of intention is rarely explicitly defined, but the fact that the results acquired are taken to constitute a “decoding” or “reading” (Haynes et al., 2007) of intentions implies that intentions are defined as processes preparing for, and causally contributing to, behavior. This presumed causal role played by intentions is discussed explicitly in various papers. For example, Haynes (2011, p. 16) warns in the discussion section of the paper that “it is unclear if the early predictive signals are also causally involved in the decision”. More explicitly, Haggard and Clark (2003, p. 696) refer to the “causal chain between intention and action”: they state that the “normal experience of intentional action includes an implicit content that the action occurred *because*, and *via* the intention that the agent had to perform it” (p. 696, emphasis in original).

However, some authors have recently argued that there is reason to doubt the existence of identifiable neurocorrelates of intentions, understood as discrete, causally efficacious mental states (Uithol et al., 2014; Burnston, 2015; Schurger and Uithol, 2015; Verbaarschot et al., 2016). Most notably, Uithol et al. (2014) have provided a thorough review showing that neural activity in the areas traditionally associated with action control (the lateral prefrontal cortex) is consistently found to be both informationally and dynamically complex: “the processes underlying action initiation and control are considerably more dynamic and context sensitive than the concept of intention can allow for”. For example, on the traditional understanding of intention, the intention to eat an apple is thought to be free from specific details such as which kind of apple to eat, where to eat it, with which movements to pick it up, and so on. However, Uithol and colleagues argue that activity in the lPFC is highly context-sensitive in that it integrates all kinds of perceptual information, a finding that undermines the idea that intentions are realized as context-free representational states. Secondly, the complexity of the neural activity underlying action control also undermines the idea that intentions are *discrete* states, in the sense that the intention initiating the action control process would be the same state as the intention that will ultimately bring the process to completion (the action into execution). In other words, so far it has proven impossible to track one specific stable state or process that guides action from beginning to end, and which thus could serve as the kind of discrete stable state an intention is supposed to be. Taken together, this picture undermines the idea that the lPFC hosts the kind of discrete higher-level neural activity that can be considered the neural instantiation of abstract, discrete intention states that causally contribute to voluntary action (Uithol et al., 2014).

Based on their analysis, Uithol et al. conclude that the notion of intention is of little value to the science of action control, and that we are in need of a reinterpretation of action control, a reinterpretation shifting “attention away from localization, as one would expect a control system of the type outlined above to be in continual and various interactions with both perceptual and motivational information” (Uithol et al., 2014, p. 137).

My aim in this paper is to build on the important insights formulated by Uithol et al., but to argue (against eliminativist conclusions one might draw on the basis of such insights) that there is no need to discard the notion of intention. Instead, I will show that neither the philosophical tradition nor our folk psychology force us to understand intentions as abstract, discrete mental states that cause our actions. Instead, I will outline an alternative Wittgensteinian approach to understanding intention, an understanding which might actually provide an interesting explanation of the findings of Uithol and colleagues. In its anti-eliminativist Wittgensteinian spirit, my approach is indebted to the general critical analysis of the relation between neuroscience and folk psychology which was developed by Bennett and Hacker (2003). In their seminal and influential book, they criticized contemporary neuroscience for generating so-called “mereological confusions” by ascribing characteristics to *parts* of human beings (namely brains) that can only be sensibly ascribed to human beings *as wholes*. For example, they argue that whereas human beings can think, argue, be angry and so on, brains cannot do any of these things (Bennett and Hacker, 2003, pp. 68–74). Although their work is clearly present as inspirational background in the current paper, my aim is not so much to add to their critical analysis but to provide a positive answer to the question what intentions are (a concept which hardly occurs in Bennett and Hacker’s analysis) and what it means to ascribe intentions to human beings.

## Intentions as Causal Mental States?

According to the traditional view, intentions are relatively abstract (high-level, amodal) representations that cause our actions. For example, my intention to make coffee is understood as a mental state involving a representation with the content “make coffee”, which is abstract in that it does not specify precisely what movements I will make in making coffee (there are various ways in which I might do this). And according to this view, it is this representational state that causally explains the fact that I walk to the kitchen and start making coffee as soon as I arrive at work. This basic understanding of what intentions are, is widely employed in both the philosophy, psychology and neuroscience of intention (Davidson, 1978; Bratman, 1999; Haggard and Libet, 2001), even by those who have developed critical views on the actual causal effectiveness of such intention states (Gollwitzer, 1999; Holton, 1999, 2003; Gollwitzer and Sheeran, 2006). In their introduction, Uithol and colleagues state that this is also the “folk psychological” understanding of intention (Uithol et al., 2014, p. 130). But is that really true? Is this how “the folk” talk and think about intentions? In this section I will argue that the folk psychology of intention is multifaceted and fluid, leaving room for various theoretical interpretations. Instead, I will show that the understanding of intentions as abstract, discrete, causally effective representational states is primarily an *academic* notion of intention, which has its sources in the causal theory of action (Davidson, 1978; Bratman, 1999; Mele, 2009) and in certain functionalist assumptions that underlie the current psychology and neuroscience of intention (see for example Haynes et al., 2007; Gallivan et al., 2011).

So how should we characterize the folk psychology of intention (and is there even such a thing)? If one starts to think about how people use terms like *intention* and *intending* in their everyday lives, one of the first things to notice is that we use them in very different ways (Anscombe, 1957). For example, we both use intention talk to describe plans for the future (*I intend to become rich one day*), and to give information about past actions (*I did not intend this to happen*). Also, data from experimental philosophy show that intention talk is often used to communicate our moral judgments. In an empirical study, Knobe (2006) showed that if John does something which has unintended but harmful side-effects on the environment we say that *John intended to harm the environment* – but when the side-effect benefits the environment, we do not say that *John intended to help the environment*. In other words, we often use terms like intention and intending to ascribe blame. Moreover, intention talk does not develop in isolation from scientific and philosophical discourses: different theories on what intentions are (more on these below), trickle down into folk psychological use.

These brief considerations suggest that there is no such thing as one distinct and clearly defined “folk psychology” of intention. This coheres with the various strands of thinking about what folk psychology is: even though philosophers disagree on the question whether the main aim of folk psychology is prediction and explanation (Churchland, 1981; Dennett, 1987), or social regulation (McGeer, 2007; Zawidzki, 2008), both traditions acknowledge that our folk psychological use of mental concepts can have various different functions (Andrews, 2015). Therefore, there seems to be no reason to think that “the folk” think of intentions as “clearly identifiable, relatively simple mental states, free from context-specific details, that are the originating causes of subsequent action planning and motor movement” (Uithol et al., 2014 p. 130). As I will show now (a point which is also mentioned by Uithol and colleagues), that definition arose from theoretical sources in both the causal theory of action and in functionalism.

The causal theory of action (Aguilar and Buckareff, 2010) was developed in analytic philosophical circles in the 1960s as a response to the then dominant view that we should explain actions by providing *reasons*, and that such explanation mainly involves redescribing the action in terms of the agent’s goals (Anscombe, 1957). In his famous paper *Actions, reasons and causes*, Davidson (1963) objected that this way of explaining actions does not suffice: as we might have various reasons for acting in a certain way, a genuine explanation of an action requires insight in *the* reason for which the agent acted. According to Davidson, the primary reason for which we act is the reason that actually *caused* the action: rational explanation thus is causal explanation. In a later paper, Davidson (1978) argued that in order to provide such causal explanations, we need not only the concept of a reason for action but also the concept of *intention* – thereby introducing the notion of intention as a representational state (with the content “I will do X”) causing our actions.

Whereas the causal theory of action is still a highly influential framework in the philosophy of action, it is also contested. Its two main challenges are firstly, that the framework has given birth to the problem of mental causation (Kim, 2000; Davidson, 2001; Robb and Heil, 2018): if reasons and intentions are mental states that make a causal difference, we should be able to understand how a mental state can intervene in the physical world. The question how this is possible still constitutes one of the main problems debated in contemporary philosophy of mind. The second challenge is Davidson’s statement that reasons and intentions only explain our actions if they caused the action *in the right way*. This was meant to exclude cases of deviant causation: cases in which our mental states cause our actions, but only in a “weird” way. The challenge is that it has turned out to be extremely difficult to provide a satisfactory account of what it would mean for mental states to cause our actions in the right way (for some attempts see Peacocke, 1979; Sehon, 1997; Stout, 2010; Hyman, 2014) – Davidson himself even concluded that it was impossible to meet this challenge (Davidson, 2001). Due to the persistence of these challenges, non-causalist approaches to action explanation have become more and more influential during the past 20 years (Baker, 1989; Thompson, 2008; Aguilar and Buckareff, 2010; Ford et al., 2011).

The ascent of the causal theory of action co-evolved with the development of the functionalist paradigm, which opposed behaviorism and built on the idea that in order to identify or demarcate mental states we should not look at their structure or at the stuff they are made of, but at the *functional roles* they play in a cognitive system (Armstrong, 1981; Fodor, 1985; Heras-Escribano, 2019). For example: states we call *intentions* play the role of securing commitment to a certain path of action. Therefore, a core element of functionalism is the idea that mental states are *multiply realizable*: in order for a certain functional state to be characterized as an intention, belief or desire, the only thing that counts is the functional role: how exactly the state is realized does not matter. However, traditional functionalism long held on to the idea that mental states should be analyzed as internal representations (Fodor, 1985). Even though functionalist assumptions can be said to underlie much of cognitive science and cognitive neuroscience, in its traditional form functionalism is just as contested as the causal theory of action. The most important opposition has been developed from within the situated cognition movement (Clark and Chalmers, 1998; Bechtel, 2009; Hutto, 2013; Varela et al., 2017), which has argued that classical functionalism does not do justice to crucial features of cognition such as its dynamic character and context-sensitivity, the interaction between cognition and other bodily processes, and the mutual interconnectedness of perception and action.

Of course, these sketchy remarks do not even come close to a comprehensive analysis of either the causal theory of action or functionalism. However, what I have tried to show is that although both paradigms are influential and fruitful on many levels, they are also vulnerable to recurring theoretical objections: the theoretical framework for making sense of intention in science is far from completed. Also, I have shown that our folk psychology leaves room for various ways to understand intentions. Based on these considerations, and especially given the bleak picture of the neuroscience of intention sketched by Uithol and colleagues, this suggests that there might be theoretical wiggle room for alternative accounts of intention.

## An Alternative Way to Think About Intention

My aim for the remainder of this paper is to outline a possible alternative understanding of intention. To most neuroscientists and psychologists, the understanding I will propose will sound impossibly radical, antinaturalist and maybe even slightly ridiculous: the view can be summarized by the statement that intention might not be “a mental state nor a combination of desires and beliefs nor anything else” (Scheer, 2004). However, I hope to show that this alternative understanding has three advantages: (1) it avoids recurrent troubles associated with both the causal theory of action and functionalism, (2) it provides a plausible interpretation of the varied nature of the folk psychology of intention and (3) it provides an interesting explanation of the absence of the types of neural correlates discussed by Uithol and colleagues.

According to the alternative perspective I propose, we use terms like “intention” and “intending” to indicate which (past, current and future) actions we commit ourselves to. This understanding of intention is based on the philosophy of psychology developed by the later Wittgenstein (1953), and it also coheres with the analysis of intention provided in Anscombe’s influential book *Intention* (Anscombe, 1957), an approach which recently experienced a comeback as one of the serious contenders in the philosophical quest to understand human action (Vogler, 2001; Thompson, 2008; Ford et al., 2011; Van Miltenburg, 2011; Tanney, 2018). I will show how this account can shed light on the various features of our folk psychological understanding of intention as described above. Also, I will show that this account would predict precisely the kind of neuroscientific findings discussed by Uithol and colleagues, and could thus provide a possible theoretical explanation of those findings.

Paradigms such as the causal theory of action and functionalism in cognitive science are grounded in the idea that explanation requires insight in underlying causal processes. According to the Wittgensteinian approach, this quest has unfortunately also led to the *reification* (understanding-as-objects) of certain aspects of our psychology that do not lend themselves to such reification. In parallel to Wittgenstein, Gilbert Ryle developed this point in vivid detail in his seminal book *The concept of mind* (Ryle, 1949). There he argues that even though philosophers of mind have set aside the Cartesian idea that mind is a distinct type of matter, they still fail to see that “mind” is a concept of a different order than the concept “matter”.

Instead, their faulty conclusion has been that mind is in fact the *usual* type of matter: that mental concepts *can*, after all, be ultimately understood in terms of physical states and processes (Ryle, 1949, p. 22). By talking about intentions in terms of states and processes, we have come to believe that what we are talking about are localized happenings taking place inside the brains of human beings. As Wittgenstein states:

“How does the philosophical problem about mental processes and states […] arise? – The first step is the one that altogether escapes notice. We talk of processes and states and leave their nature undecided. Sometime perhaps we shall know more about them – we think. But that is just what commits us to a particular way of looking at the matter. […] (The decisive movement in the conjuring trick has been made, and it was the very one that we thought quite innocent).” (*Philosophical Investigations*, 308).

However, from a Wittgensteinian/Rylean perspective, there are several philosophical reasons for doubting that mental concepts are realized as discrete states or processes in the brain. In Ryle’s chapter in *The concept of mind* on the will, he argues forcefully that “having the intention to do something” (Ryle mostly uses the term volition here) cannot meaningfully refer to the occurrence of any kind of episode of intending or willing: “No one ever says such things as that at 10 a.m. he was occupied in willing this or that, or that he performed five quick and easy volitions and two slow and difficult volitions between midday and lunch-time” (Ryle, 1949, p. 64). Similar ideas are found in Wittgenstein’s Philosophical Investigations, which have been developed in illuminative and concrete detail by Scheer (2004). As Scheer argues, we do not ascribe intensity nor temporal duration to intentions, something which would be a natural thing to do for mental states. We do not say things like “Charles” intention to go to the party was twice as strong as the intention of his wife’s. Nor do we say: “from 5 PM to 8 PM he intended to go to the party, but then he revised his intention”. A second argument developed by Scheer is that whereas the starting point of our thinking about intention is the idea that intending is characterized by commitment, this is actually difficult to maintain on the view that intentions are causally efficacious mental states. The connection between intention and commitment seems to be that when we intend to wash the car, this means that we *believe* we will wash the car, as long as no overriding reasons to change plans come up. However, on the causal view, the self-ascription of an intention becomes *a mere prediction* concerning the causal efficacy of the mental states one takes oneself to have (Scheer, 2004). This leaves room for the possibility (discussed by Bratman (1987)) that one intends to wash the car even though one does not believe that one will wash the car, for instance because one knows one is prone to forgetting such chores. This possibility severes the link between intention and commitment Scheer, and many others, consider to be an essential feature of the concept of intention.

So if intentions are *not* discrete states or processes that are realized in the brain, what are they? Scheer seems to point at something important when he introduces the notion of commitment; however, he does not provide any arguments showing *why* commitment would be an important feature of intention. Taking Scheer’s suggestions a step further, my proposal is that the connection between intention and commitment is crucial because intention plays certain kinds of roles in our folk psychology, some of which cannot be covered by the causalist/functionalist understanding of intentions as discrete mental states. To be more specific: when we talk about intentions in our everyday lives we express a first-person commitment towards an action; and when others ascribe intentions to us, they thus ascribe such commitment to us. Now the crucial point is that such commitment involves adopting *a normative stance*: we do such expressing and ascribing in order to determine what we can expect from others and from ourselves (Moran, 2001). This suggests that the folk psychological notion of intention is thus at least partly a normative one: we use it not only to predict or explain behavior, but also to evaluate and to regulate it. This view that social regulation is one of the core functions of folk psychology has recently been developed in more philosophical detail by authors such as McGeer (2007, 2008, 2015) and Zawidzki (2008). They argue that often, when we ascribe folk psychological mental states to others or to ourselves, we are “engaged in the activity of moulding behaviour – cajoling, encouraging, reprimanding, promising and otherwise giving ourselves over to the task of producing comprehensible patterns of well-behaved agency in ourselves and others” (McGeer, 2007, p. 149).

Such an understanding of intention cannot be covered by the causalist/functionalist paradigm because *in so far as* intentions are expressions of normative expectations, they do not refer to previous causal states. In fact: they do not refer to “anything at all”: this is how one should understand Scheer’s dictum that an intention is not “a mental state nor a combination of desires and beliefs nor anything else” (Scheer, 2004).

However, this raises the question: what’s the difference between Charles, whom we take to have the intention to go to the party next week, and Jane, whom we do not believe to have any such intention? Even if one accepts that the way we talk about intentions is often a way to express our normative expectations, such expectations are only warranted in so far as the agent *is indeed committed*. So we need to answer the question: what makes the ascription of an intention (by others or by oneself) legitimate? It cannot be the case that just because one says one has an intention, one thereby necessarily has it. As Wittgenstein already emphasized: “the most explicit expression of intention is by itself insufficient evidence of intention” (Wittgenstein, 1953, p. 641). As I will explain below, the Wittgensteinian answer (which is in a different form also found in Dennett, 1991) is to understand the ascribing of intentions as a form of pattern recognition, leading to the criterion that someone truly has an intention *if and only if* a certain pattern is present in the world.

## Intentions and Patterns

According to the Wittgensteinian approach, the statement that “Charles has the intention to go to the party next week” is true in so far as a certain pattern of phenomena is present in the world; a pattern that is extended in both space *and* time (Wittgenstein, 1982; Ter Hark, 2001). The notion of a pattern bears important similarities to dispositional accounts of mental states (Ryle, 1949; Schwitzgebel, 2013; Tumulty, 2014), with the important difference that the Wittgensteinian approach is manifestly non-metaphysical: where most dispositionalists *identify* intentions with dispositional patterns^1^, Wittgensteinians claim that in saying “Charles intends to go to the party” we mean to say that a certain pattern exists in the world; but this pattern is not itself the intention (Ter Hark, 2001). As we will see below, the latter point is important because it shows that from a Wittgensteinian perspective, intentions do not cause anything.

To illustrate what such a pattern indicated by intention ascriptions might entail, I will give a few examples of relevant “elements” of such a pattern: Charles has the intention to go to the party in so far as he will make preparations for the party, will not make conflicting appointments for that evening, experiences feelings of anticipation, and answers questions of others (“did you buy a gift?”) in certain ways. From this sketchy list, several things immediately become clear. First of all, the pattern on the basis of which we say that Charles has a certain intention is vague and consists of a wide variety of different elements, none of which is in itself crucial for the ascription of intention. In most situations, certain elements will be missing (for example, Charles might make a conflicting appointment for that evening because he checks the wrong date in his calendar). However, the pattern should be present to a critical extent in order for us to legitimately ascribe an intention. Just as with dispositional accounts of belief, there will be a grey area in which agents have “in-between” intentions, to borrow Eric Schwitzgebel’s notion (Schwitzgebel, 2002, 2013). In such intermediate cases (let us say for example that Charles keeps saying that he will go to the party, while he does not make any preparations and keeps forgetting about it), there is no objective fact of the matter regarding the question whether Charles intends to go to the party or not: it is indeterminate. Several Wittgensteinian philosophers have argued that such implications do not constitute a weakness in the theory, but that indeterminacy is an inherent feature of the mental (McDowell, 1992; Ammereller, 2001; Ter Hark, 2001; Child, 2011).

The fact that the pattern which makes it true that someone has a certain intention is extended also in time, implies that *only over time* will it become clear whether someone has an intention or not. Wittgenstein makes a similar remark about the concept of expectation:

“If I say: “I have been expecting him all day”, “expect” here doesn’t mean a persistent condition including as ingredients the person expected and his arrival, in the way that a dough may contain flour, sugar and eggs mixed into a paste. What constitutes expectation is a series of actions, thoughts and feelings.” (Wittgenstein, 1974, p. 141)

This also implies that it is impossible to have an intention over a very brief period of time, Wittgenstein makes this point about mental concepts in general, and gives the following example of love: “Could someone have a feeling of ardent love and hope for the space of a second – *no matter what* preceded or followed this second? – What is happening now has significance – in these surroundings. The surroundings give it its importance” (Wittgenstein, 1953, p. 583). This emphasis on the temporal extendedness of intention might be surprising: when comparing different mental concepts, precisely the phenomenon of intention seems to be a pretty local phenomenon, taking place somewhere in between a decision and an action. However, Wittgenstein emphasizes that we can actually only make sense of someone’s intentions when we look at the person in the context of his past (how this person decided and acted before) and his future (what this person did and said later).

For anyone interested in the neuroscience of intention, this account of how to understand intentions immediately raises the question: but does not all this imply that intentions just do not exist, that intention-talk is just that – only a matter of speaking? It is true that in a strong sense, according to the Wittgensteinian approach intentions do not exist: they are not *things*, they are not real in the way that chairs and stars and molecules are real. However, intentions *do* exist in the sense that there are often *real facts of the matter* regarding what someone intends. In this sense, Wittgensteinians can still say that people *have* intentions – but having intentions is more like “having a quick temper” than like “having a car”. It should be emphasized that the approach outlined here is not a form of interpretivism, which would claim that there is no truth about the intentions ascribed independent of the ascribing (Mölder, 2010). To the contrary, ascribing to Charles an intention “to go to the party” is recognizing a pattern that is already there, independent of the ascription. This also suggests that people can be misled about their own intentions: merely saying that you intend to mow the lawn does not make it so. Whether or not this *truly* is your intention depends on whether the pattern is present to a sufficient extent.

However, for most “scientifically oriented” philosophers this still seems a threateningly anti-naturalist position to adopt. As said, Dennett came close to developing a form of “pattern realism” in his early work (Dennett, 1991; Ter Hark, 2001). However, it was central to Dennett’s understanding of concepts such as intention that intentions were *causally effective states* (Slors, 2007). As a result, Dennett argued that a mental state such as an intention should be *identified* with a pattern, and that it is the pattern itself that is causally efficacious (where the pattern could be further explained in terms of underlying physical states and processes). In order to make this work, Dennett employed a temporally and spatially local notion of patterns which are “quite readily discernible to the naked human eye” (Dennett, 1991, p. 33), a point which invited strong criticism: “If there are “visible patterns” of bodily movements that constitute believing that Clinton is President, I, for one, am totally ignorant of what they are” (Nelkin, 1994, p. 63). The Wittgensteinian response to Dennett’s approach would be to argue that because intention ascriptions refer to patterns that are extended in both time and space, it becomes meaningless to say that the intention is the pattern, because then our mental states would no longer “belong to us” in any meaningful sense. The more plausible conclusion, for Wittgensteinians, thus comes down to the claim that intentions are not mental states at all.

But does not this overlook one of the most important features of the concept of intention, namely that the commitment they express plays a guiding role in human action? Although the scope of this paper does not allow an extensive treatise on causation, the Wittgensteinian answer to this question boils down to saying that indeed, if intentions are not mental states, they cannot cause anything either (Scheer, 2004). This means that in order to make sense of human action, the Wittgensteinian understanding of intention requires a notion of causation that can ascribe causal powers to human agents as such. Now of course, in itself this is not a satisfying answer at all as it only solves the problem of causation-by-states by positing an equally contested form of causation-by-agents. Nevertheless it is important to emphasize that a Wittgensteinian approach does not leave room for causation-by-mental-states but, on a more positive note, that there are actually interesting recent attempts to develop views on causation that *do* offer possibilities for providing a Wittgensteinian answer to how intending agents could make things happen (see for example Mayr, 2011; Alvarez, 2013; Jacobs and O’Connor, 2013).

In the next section we will return to the question whether and how the patterns that legitimize intention ascriptions could possibly be objects of (neuro)scientific investigation.

## Implications for the Neuroscience of Intention

Even though the philosophical view outlined in the previous section might sound impossibly radical to many psychologists and neuroscientists working on intention, one important reason to discuss this view is that it actually provides an interesting explanation for the neuroscientific findings discussed by Uithol et al. (2014). As discussed in the introduction, their conclusion was that brain processes underlying action control are too dynamic to count as possible neurocorrelates of a state like intention. Whereas their own suggestion was that intention might not be a useful concept for the science of action control, my proposal here is that both the philosophical literature on intention and its folk psychological characteristics leave room for different notions of intention. I argued that we might consider the possibility that the concept of intention does not refer to any identifiable state or set of underlying processes. If this is a plausible account, it would no longer be surprising that neuroscience has so far not delivered a clear picture of neural activity realizing any state of intention. In other words: maybe we have not found neuroscientific evidence of the existence of such states because such states do not exist.

However, would not such a conclusion also imply that there is no such thing as the neuroscience of intention? As said, seen from a Wittgensteinian perspective, we use the concept of intention to express normative expectations related to commitment, where such expressions refer to the existence of certain (variable and fluid) patterns in the world. The question then becomes: can we investigate such patterns in a scientific manner? The most straightforward answer is that precisely because of their variability and fluidity, such patterns do not seem to lend themselves to scientific analysis. After all, such patterns constitute “regularities, but not so regular as to be describable in terms of rules” (Johnston, 2002) – while the aim of cognitive science seems to be precisely to determine and analyze *rules* underlying phenomena such as perception, cognition and action. To complicate things further, the patterns consist of elements that belong to various “levels of explanation”: for example, the pattern referred to by the claim *John intends to mow the lawn* might consist of speech acts such as “I think I will get it done tonight!”, certain subjective feelings of determination and the fact that his friends will not wait for him when they leave for a football match. It is hard to think of any specific science that could take all such elements and others into its stride.

So in any direct sense, the Wittgensteinian way to understand intention does not leave much room for a (neuro)science *of intention*. However, I think the Wittgensteinian perspective opens up at least three possible routes of scientific investigation. First, the patterns involved in intention-talk evidently also include physical phenomena – one might think of basic phenomena like arousal and attention. And given that neuroscience is one of the core sciences studying the processes and mechanisms underlying such phenomena, it can be expected to provide novel insights in what forming, maintaining and executing intentions might involve in terms of phenomena like attention and arousal.

Secondly, as already mentioned, there has been a surge in situated cognition approaches, manifesting itself both in theoretical and in empirical work (Clark and Chalmers, 1998; Bechtel, 2009; Hutto, 2013; Varela et al., 2017). Because these approaches understand cognition in general to be embodied, embedded, extended and/or enactive, they have an inherent interest in the question how the scope of cognitive and neuroscientific analysis could be widened beyond the brain and often also beyond the body (Silberstein and Chemero, 2012; Van Orden and Stephen, 2012; Favela and Martin, 2017). This fast-developing field could thus provide invaluable methodological tools for determining to what extent a cognitive and neuroscientific analysis of the kind of complex patterns involved in intention-talk might become possible in the future.

Finally, network theory has recently established itself as an important methodological approach within the discipline of psychometrics (Borsboom and Cramer, 2013; Schmittmann et al., 2013; Dalege et al., 2016). This approach models psychological constructs as networks of causally interconnected nodes, and as such it might prove highly relevant for the scientific investigation of the kind of patterns involved in having intentions. Because the network approach has been developed as a *psychometric* approach, it is highly open with regard to the types of phenomena that can be integrated as nodes in such networks, and does not impose restrictions on the level at which phenomena are described or explained. This suggests that insofar as it would be possible to specify the phenomena that belong to the pattern we recognize when we ascribe intentions, and in so far as these phenomena could be quantitatively measured, it would be possible to construct a network model of such a pattern, and to analyze the pattern accordingly. Whereas the current state of network science clearly does not yet allow for any such a model, it would be worthwhile to examine its potential for our future understanding of intention.

## Author Contributions

The author confirms being the sole contributor of this work and has approved it for publication.

### Conflict of Interest Statement

The author declares that the research was conducted in the absence of any commercial or financial relationships that could be construed as a potential conflict of interest.

## Footnotes

^1^Whether this really applies to Ryle’s position is a matter of debate. Whereas Wittgenstein and Ryle definitely have similar views on what is wrong with the mainstream idea that intentions are realized in physical states, it is less clear to what extent their ‘positive’ accounts of mental concepts overlap. Although some consider Ryle to defend a crude form of behaviorism, others hold that he should be interpreted as defending an anti-metaphysical dispositional account, similar to Wittgenstein’s view (Park, 1994; Benham, 2000). To unravel Ryle’s position and its relation to Wittgenstein’s would require a separate investigation; therefore the analysis here focuses on Wittgenstein’s account, which is straightforwardly antimetaphysical.

## References

[ref1] Aguilar J. H. Buckareff A. A. (2010). Causing human actions: New perspectives on the causal theory of action. (Boston: MIT Press).

[ref2] Alvarez M. (2013). “VI—Agency and two-way powers” in Proceedings of the Aristotelian society. 113, 101–121.

[ref3] Ammereller E. (2001). “Wittgenstein on intentionality” in Wittgenstein: A critical reader. ed. Glock H.-J. (Oxford: Blackwell Oxford), 59–93.

[ref4] Andrews K. (2015). Pluralistic folk psychology and varieties of self-knowledge: an exploration. Philos. Explor. 18, 282–296. 10.1080/13869795.2015.1032116

[ref5] Anscombe G. E. M. (1957). Intention. (Cambridge: Harvard University Press).

[ref6] Armstrong D. M. (1981). “The causal theory of the mind” in Mind and cognition. A reader. (Oxford: Blackwell), 80–87.

[ref7] Baker L. R. (1989). Instrumental intentionality. Philos. Sci. 56, 303–316. 10.1086/289489

[ref8] Bechtel W. (2009). “Explanation: Mechanism, modularity, and situated cognition” in The Cambridge handbook of situated cognition, eds. Aydede M. Robbins P. (Cambridge: Cambridge University Press), 155–170.

[ref9] Benham B. (2000). Ryle and the para-mechanical. Southwest Philos. Stud. 22, 10–18.

[ref10] Bennett M. R. Hacker P. M. S. (2003). Philosophical foundations of neuroscience. Vol. 79 (Oxford: Blackwell).

[ref11] Bisley J. W. Goldberg M. E. (2010). Attention, intention, and priority in the parietal lobe. Annu. Rev. Neurosci. 33, 1–21. 10.1146/annurev-neuro-060909-152823, PMID: 20192813 PMC3683564

[ref12] Blakemore S.-J. Decety J. (2001). From the perception of action to the understanding of intention. Nat. Rev. Neurosci. 2:561. 10.1038/35086023, PMID: 11483999

[ref13] Borsboom D. Cramer A. O. (2013). Network analysis: an integrative approach to the structure of psychopathology. Annu. Rev. Clin. Psychol. 9, 91–121. 10.1146/annurev-clinpsy-050212-185608, PMID: 23537483

[ref14] Bratman M. E. (1987). Intention, plans, and practical reason. (Chicago: University of Chicago Press).

[ref15] Bratman M. E. (1999). Faces of intention: Selected essays on intention and agency. (Cambridge: Cambridge University Press).

[ref16] Burnston D. C. (2015). Perceptual context and the nature of neural function. (San Diego: University of California).

[ref17] Child W. (2011). Wittgenstein. (Abingdon: Routledge).

[ref18] Churchland P. M. (1981). Eliminative materialism and the propositional attitudes. J. Philos. 78, 67–90.

[ref19] Clark A. Chalmers D. (1998). The extended mind. Analysis 58, 7–19.

[ref20] Dalege J. Borsboom D. van Harreveld F. van den Berg H. Conner M. van der Maas H. L. J. (2016). Toward a formalized account of attitudes: the causal attitude network (CAN) model. Psychol. Rev. 123, 2–22. 10.1037/a0039802, PMID: 26479706

[ref21] Davidson D. (1963). Actions, reasons, and causes. J. Philos. 60, 685–700. 10.2307/2023177

[ref22] Davidson D. (1978). “Intending” in Philosophy of history and action. ed. Yovel Y. (Dordrecht: Springer), 41–60.

[ref23] Davidson D. (2001). Essays on actions and events. Vol. 1 (USA: Oxford University Press).

[ref24] Dennett D. C. (1987). The intentional stance. (Cambridge: The MIT Press).

[ref25] Dennett D. C. (1991). Real patterns. J. Philos. 88, 27–51. 10.2307/2027085

[ref26] Favela L. H. Martin J. (2017). “Cognition” and dynamical cognitive science. Mind. Mach. 27, 331–355. 10.1007/s11023-016-9411-4

[ref27] Fodor J. A. (1985). Fodor’s guide to mental representation: the intelligent auntie’s vade-mecum. Mind 94, 76–100.

[ref28] Ford A. Hornsby J. Stoutland F. (Eds.) (2011). Essays on Anscombe’s intention. (Cambridge, Mass: Harvard University Press).

[ref29] Gallivan J. P. McLean D. A. Valyear K. F. Pettypiece C. E. Culham J. C. (2011). Decoding action intentions from preparatory brain activity in human parieto-frontal networks. J. Neurosci. 31, 9599–9610. 10.1523/JNEUROSCI.0080-11.2011, PMID: 21715625 PMC6623162

[ref30] Gollwitzer P. M. (1999). Implementation intentions: strong effects of simple plans. Am. Psychol. 54, 493–503. 10.1037/0003-066X.54.7.493

[ref31] Gollwitzer P. M. Sheeran P. (2006). Implementation intentions and goal achievement: a meta-analysis of effects and processes. Adv. Exp. Soc. Psychol. 38, 69–119. 10.1016/S0065-2601(06)38002-1

[ref32] Haggard P. Clark S. (2003). Intentional action: conscious experience and neural prediction. Conscious. Cogn. 12, 695–707. 10.1016/S1053-8100(03)00052-7, PMID: 14656511

[ref33] Haggard P. Libet B. (2001). Conscious intention and brain activity. J. Conscious. Stud. 8, 47–64.

[ref34] Haynes J.-D. (2011). Decoding and predicting intentions. Ann. N. Y. Acad. Sci. 1224, 9–21. 10.1111/j.1749-6632.2011.05994.x, PMID: 21486293

[ref35] Haynes J.-D. Sakai K. Rees G. Gilbert S. Frith C. Passingham R. E. (2007). Reading hidden intentions in the human brain. Curr. Biol. 17, 323–328. 10.1016/j.cub.2006.11.072, PMID: 17291759

[ref36] Heras-Escribano M. (2019). The philosophy of affordances. (Cham: Palgrave Macmillan).

[ref37] Holton R. (1999). Intention and weakness of will. J. Philos. 96, 241–262. 10.2307/2564667

[ref38] Holton R. (2003). “How is strength of will possible?” in Weakness of will and practical irrationality. eds. Tappolet C. Stroud S. (Oxford: Clarendon Press), 39–67.

[ref39] Hutto D. D. (2013). Psychology unified: from folk psychology to radical enactivism. Rev. Gen. Psychol. 17:174. 10.1037/a0032930

[ref40] Hyman J. (2014). Desires, dispositions and deviant causal chains. Philosophy 89, 83–112. 10.1017/S0031819113000685

[ref41] Jacobs J. D. O’Connor T. (2013). “Neo-Aristotelian metaphysics” in Mental causation and ontology. eds. Gibb S. C. Lowe E. J. Ingthorsson R. D. (Oxford: Oxford University Press), 173–192.

[ref42] Johnston P. (2002). Wittgenstein: Rethinking the inner. (Abingdon: Routledge).

[ref43] Kim J. (2000). “The mind-body problem: where we now are” in Mind in a physical world: An essay on the mind-body problem and mental causation. (Boston: MIT Press).

[ref44] Knobe J. (2006). The concept of intentional action: a case study in the uses of folk psychology. Philos. Stud. 130, 203–231. 10.1007/s11098-004-4510-0

[ref45] Libet B. (1985). Unconscious cerebral initiative and the role of conscious will in voluntary action. Behav. Brain Sci. 8, 529–539.

[ref46] Libet B. Gleason C. A. Wright E. W. Pearl D. K. (1983). Time of conscious intention to act in relation to onset of cerebral activity (readiness-potential) the unconscious initiation of a freely voluntary act. Brain 106, 623–642. 10.1093/brain/106.3.623, PMID: 6640273

[ref47] Mayr E. (2011). Understanding human agency. (Oxford: Oxford University Press).

[ref48] McDowell J. (1992). Meaning and intentionality in Wittgenstein’s later philosophy. Midwest Stud. in Philos. 17, 40–52. 10.1111/j.1475-4975.1992.tb00141.x

[ref49] McGeer V. (2007). “The regulative dimension of folk psychology” in Folk psychology re-assessed. eds. Hutto D. Radcliffe M. (Dordrecht: Springer), 137–156.

[ref50] McGeer V. (2008). The moral development of first-person authority. Eur. J. Philos. 16, 81–108. 10.1111/j.1468-0378.2007.00266.x

[ref51] McGeer V. (2015). Mind-making practices: the social infrastructure of self-knowing agency and responsibility. Philos. Explor. 18, 259–281. 10.1080/13869795.2015.1032331

[ref52] Mele A. R. (2009). Effective intentions: The power of conscious will. (Oxford: Oxford University Press).

[ref53] Mölder B. (2010). Mind ascribed: An elaboration and defence of interpretivism. Vol. 80 (Amsterdam: John Benjamins Publishing).

[ref54] Moran R. (2001). Authority and estrangement: an essay on self-knowledge. (Princeton: Princeton University Press).

[ref55] Nelkin N. (1994). Patterns. Mind Lang. 9, 56–87. 10.1111/j.1468-0017.1994.tb00216.x

[ref56] Park S. M. (1994). Reinterpreting Ryle: a nonbehavioristic analysis. J. Hist. Philos. 32, 265–290. 10.1353/hph.1994.0043

[ref57] Peacocke C. (1979). Deviant causal chains. Midwest Stud. in Philos. 4, 123–155. 10.1111/j.1475-4975.1979.tb00375.x

[ref58] Robb D. Heil J. (2018). “Mental causation” in The Stanford Encyclopedia of Philosophy (Winter 2018 edition). ed. Zalta E. N.. https://plato.stanford.edu/archives/win2018/entries/mental-causation/

[ref59] Ryle G. (1949). The concept of mind. (Chicago: University of Chicago Press).

[ref60] Scheer R. (2004). The ‘mental state’ theory of intentions. Philosophy 79, 121–131. 10.1017/S0031819104000087

[ref61] Schmittmann V. D. Cramer A. O. J. Waldorp L. J. Epskamp S. Kievit R. A. Borsboom D. (2013). Deconstructing the construct: a network perspective on psychological phenomena. New Ideas Psychol. 31, 43–53. 10.1016/j.newideapsych.2011.02.007

[ref62] Schurger A. Uithol S. (2015). Nowhere and everywhere: the causal origin of voluntary action. Rev. Philos. Psychol. 6, 761–778. 10.1007/s13164-014-0223-2

[ref63] Schwitzgebel E. (2002). A phenomenal, dispositional account of belief. Noûs 36, 249–275. 10.1111/1468-0068.00370

[ref64] Schwitzgebel E. (2013). “A dispositional approach to attitudes: thinking outside of the belief box” in New essays on belief: Constitution, content and structure. ed. Nottelmann N. (Dordrecht: Springer), 75–99.

[ref65] Sehon S. R. (1997). Deviant causal chains and the irreducibility of teleological explanation. Pac. Philos. Q. 78, 195–213. 10.1111/1468-0114.00035

[ref66] Silberstein M. Chemero A. (2012). Complexity and extended phenomenological-cognitive systems. Top. Cogn. Sci. 4, 35–50. 10.1111/j.1756-8765.2011.01168.x, PMID: 22253176

[ref67] Slors M. V. (2007). Intentional systems theory, mental causation and empathic resonance. Erkenntnis 67, 321–336. 10.1007/s10670-007-9074-x

[ref68] Stout R. (2010). “Deviant causal chains” in A companion to the philosophy of action. eds. O’Connor T. Sandis C. (Singapore: Wiley-Blackwell), 159–165.

[ref69] Tanney J. (2018). Remarks on the “thickness” of action description: with Wittgenstein, Ryle, and Anscombe. Philos. Explor. 21, 170–177. 10.1080/13869795.2017.1421698

[ref70] Ter Hark M. (2001). “Wittgenstein and Dennett on patterns” in Wittgenstein and contemporary philosophy of mind. (London: Palgrave Macmillan), 85–103.

[ref71] Thompson M. (2008). Life and action. (Boston: Harvard University Press).

[ref72] Tumulty M. (2014). Managing mismatch between belief and behavior. Pac. Philos. Q. 95, 261–292. 10.1111/papq.12032

[ref73] Uithol S. Burnston D. C. Haselager P. (2014). Why we may not find intentions in the brain. Neuropsychologia 56, 129–139. 10.1016/j.neuropsychologia.2014.01.010, PMID: 24462950

[ref74] Van Miltenburg N. (2011). Practical knowledge and foreseen side effects. J. Ethics Soc. Philos. 6, 1–7. 10.26556/jesp.v6i1.147

[ref75] Van Orden G. Stephen D. G. (2012). Is cognitive science usefully cast as complexity science? Top. Cogn. Sci. 4, 3–6. 10.1111/j.1756-8765.2011.01165.x, PMID: 22253173

[ref76] Varela F. J. Thompson E. Rosch E. (2017). The embodied mind: Cognitive science and human experience. (Cambridge: MIT Press).

[ref77] Verbaarschot C. Haselager P. Farquhar J. (2016). Detecting traces of consciousness in the process of intending to act. Exp. Brain Res. 234, 1945–1956. 10.1007/s00221-016-4600-1, PMID: 26920393 PMC4893062

[ref78] Vogler C. (2001). “Anscombe on practical inference” in Varieties of practical reasoning. ed. Millgram E. (Boston: Harvard University Press), 437–464.

[ref79] Wittgenstein L. (1953). Philosophical Investigations. (G. E. M. Anscombe, Vert.). (Oxford: Blackwell).

[ref80] Wittgenstein L. (1974). Philosophical Grammar. eds. Rhees R. Kenny A. (Oxford: Basil Blackwell).

[ref81] Wittgenstein L. (1982). Last writings on the philosophy of psychology. Vol. II, eds. von Wright G. H. Nyman H. Luckhardt C. G. Maximilian A. E., (Chicago: University of Chicago Press).

[ref82] Zawidzki T. W. (2008). The function of folk psychology: mind reading or mind shaping? Philos. Explor. 11, 193–210. 10.1080/13869790802239235

